# Development of a New Handheld Device for Measuring Photosynthetic Carbon Dioxide Assimilation in Plant Leaves

**DOI:** 10.3390/plants15121888

**Published:** 2026-06-18

**Authors:** Elizaveta Kozlova, Denis Zbruev, Alexey Baburkin, Ekaterina Sukhova, Vladimir Sukhov

**Affiliations:** 1 Department of Biophysics, N.I. Lobachevsky State University of Nizhny Novgorod, 603950 Nizhny Novgorod, Russia; denis9159597861@yandex.ru (D.Z.); aleksejbaburkin@gmail.com (A.B.); n.catherine@inbox.ru (E.S.); vssuh@mail.ru (V.S.); 2 Department of Mathematical Software and Supercomputing Technologies, N.I. Lobachevsky State University of Nizhny Novgorod, 603950 Nizhny Novgorod, Russia

**Keywords:** assimilation measurement, crop health, early stress detection, hand-held devices, new phenotyping methods, smart agriculture

## Abstract

With increasing constraints on extensive farming—including soil degradation, salinisation and more frequent climatic anomalies—the development of ‘smart’ agriculture requires the integration of affordable, non-invasive methods for monitoring the physiological state of plants. A key indicator for assessing productivity and the early detection of stress is the rate of photosynthetic CO_2_ assimilation (*A*); however, widely available commercial gas analysers are characterised by high cost, technical complexity and considerable weight, which limits their use in large-scale field studies. Here, a new handheld system for measuring assimilation was developed and tested, based on the accumulative principle of recording changes in CO_2_ concentration using simple infrared sensors and without maintaining a constant air flow around the leaf. A comparison was carried out between a prototype of the developed system and a commercial gas analyser when measuring leaf assimilation under irrigation and simulated drought conditions. The results demonstrated the consistency of the readings from the two systems. The developed system is characterised by its compact size, low cost, and the absence of moving parts and consumables. The proposed system has the potential to be effective for large-scale screening tasks and rapid diagnosis of stress-induced changes; it represents a promising, affordable tool for addressing applied tasks in precision agriculture, environmental monitoring and physiological research.

## 1. Introduction

Today, agriculture forms the very foundation of the global food industry, playing a significant role in the economies of many countries. At the same time, extensive farming methods are facing an increasing number of limitations due to the need to improve crop cultivation efficiency and reduce environmental impact. The development of intensive approaches, based on the active application of innovative technologies in agriculture, is the primary way to increase crop yields and maintain plant productivity under adverse environmental conditions [1,2,3,4]. Today, the development of ‘smart’ agriculture can be described as the primary response to such significant challenges as soil erosion and degradation resulting from the excessive use of fertilisers and pesticides, the salinisation of arable land, a general reduction in the availability of fresh water, as well as an increase in the frequency of adverse climatic events (drought, extreme high and low temperatures, excessive precipitation) resulting from global climate change [1,2,3,4].

One of the most significant parts of modern agriculture is the continuous or periodic monitoring of crop health, with the aim of assessing their fertiliser requirements and detecting early signs of stress that may arise due to abiotic and biotic stressors [2,4,5]. The traditional method for addressing this task is visual observation of plants, which can be carried out by manual crop monitoring (scouting). However, such observation is a labour-intensive and relatively slow process, requiring researchers to possess a sufficient level of expert knowledge in assessing plant condition and identifying damage [1,4,6]. Furthermore, visual assessment is not sufficiently effective for detecting early changes in plants under adverse conditions [5,7].

The development of devices and systems for the quick and non-invasive measurement of key plant parameters—including the detection of stress-induced changes in physiological indicators—is of crucial importance [8,9]. Among non-invasive methods, one can distinguish between contact (requiring direct contact with the plant), proximal (measuring plant parameters at a moderate distance from the object) and remote (measuring parameters at significant distances from the object) methods [10]. Among the most significant parameters used in plant condition monitoring are morphometric parameters [11], including the 3D structure of shoots and roots [12] and general features of the plant’s appearance, which may be analysed using computer vision [4,13], electrophysiological status [14], transpiration rate [15] and plant temperature [7,15]. Photosynthetic indices are very important among these parameters [12,15,16], as photosynthesis forms the basis of plant production and is highly sensitive to the effects of stressors [17,18]. In particular, the assessment of photosynthetic parameters is of great importance for yield forecasting and early detection of the impact of adverse conditions on plants (lack of nutrition, action of abiotic stressors, diseases, pest attacks, etc.) [7]. In agriculture, the results of such assessments enable timely protective measures to be taken, thereby helping to safeguard the crop [18]. Furthermore, measuring photosynthetic parameters plays a significant role in environmental monitoring and is a key method in applied and fundamental research into plant functioning under various conditions [4,6].

It is well known that the widely used methods for assessing photosynthesis are based on measurements of chlorophyll fluorescence (primarily PAM fluorimetry and the OJIP test) [18] and light absorption by photosystem I [18,19,20], the study of reflected light characteristics (primarily analysis of the photochemical reflectance index [21,22,23]), measurements of carbon dioxide and oxygen exchange in the open air flux [24], studies of dry matter accumulation [25], electrochemical approaches [24,25] and so on. In this context, methods for measuring the photosynthetic CO_2_ assimilation of leaves and whole plants (*A*) are especially important, as they serve as key tools for assessing the productivity of plant organisms under various conditions [6,26]. Furthermore, the initial response of plants to most stress factors (drought, high temperatures, salinity, pathogens, nutrient deficiency and others) manifests as a reduction in CO_2_ photosynthetic assimilation [27], which allows one to use measurements of this assimilation to identify the effects of stressors on plants.

Modern methods for analysing the rate of CO_2_ photosynthetic assimilation in plants and plant leaves operate in real time and are based on the use of specialised commercial systems [28,29], which ensure a constant air flow around the leaf (or whole plant), continuous measurement of changes in CO_2_ concentration within this flow using high-precision infrared CO_2_ sensors, and the calculation of key gas exchange parameters. The most popular commercial systems of this type include the Li-6800 and older models (Li-Cor, Lincoln, NE, USA), GFS-3000 (Walz GmbH, Effeltrich, Germany), CIRAS-4 (PPSystems, Hitchin, UK), LCA4 and older models (ADC-Biosciences, Hoddesdon, UK) and others. Such systems are highly sensitive to changes in CO_2_ concentration (down to 0.01 ppm) and have a high measurement frequency (up to 1 Hz), which allows for continuous monitoring of photosynthetic CO_2_ assimilation, including the detection of rapid changes; they are also capable of *E* (transpiration) measurements. Commercial systems are available in various configurations, including portable versions designed for field measurements [28,29]. It should be noted, however, that such systems are technically complex (in particular, they require a mechanical air flow maintenance system and high-precision sensors), contain moving parts (which increases the likelihood of breakdowns) and are relatively heavy (which makes their use in the field difficult). This complexity also results in the relatively high cost of commercial systems. These characteristics significantly limit the application of commercial gas analysers and highlight the need to develop simpler and more affordable systems for measuring plant gas exchange [30].

As an alternative to measuring CO_2_ in the air flow passing around a leaf, accumulative methods for determining the rate of photosynthetic CO_2_ assimilation can be used. Such methods have been known for quite some time, but they are few in number and are based on measuring the long-term (at least tens of minutes) accumulation of the products of photosynthesis by the leaf. Such methods for determining the photosynthetic rate often rely on measuring the change in CO_2_ content over a specified time interval in a flask containing a photosynthesising plant or part of it. The assimilation rate can be determined by a radioisotopic method based on the uptake of ^14^CO_2_ [31,32] or by titrating the residual CO_2_ in the flask with acid, after it has been bound with alkali [33,34]. It is important to note that classical variants of accumulative methods do not require specialised, expensive equipment; however, they are slow and have low accuracy, and in some cases require object destruction, which limits their application.

At present, the analysis of accumulative changes in CO_2_ concentration, related to photosynthetic assimilation, under light conditions can also be carried out using infrared CO_2_ concentration sensors [32,35,36,37,38]. The magnitude of accumulative changes in CO_2_ concentration must be many times greater than the changes in CO_2_ concentration under conditions of continuous air flow around the sample, which is implemented in modern commercial systems. The presence of a large signal allows the use of simpler infrared CO_2_ sensors. Currently, there have been only a few attempts to measure a plant’s assimilation based on the analysis of accumulative changes in CO_2_ concentration and the use of infrared CO_2_ sensors [35,36]; however, these are based on studies of whole plants in a special chamber (i.e., they may not be applicable in field conditions and are only applicable to plants that are not very large in size). Closed systems for analysing assimilation rates using infrared sensors are similar in design; these employ pumps to ensure an air flow from the leaf to the infrared gas analyser and beyond [32,37,38]. Thus, the use of the accumulative method for measuring photosynthetic CO_2_ assimilation for handheld devices is currently extremely limited, but has significant potential.

From the perspective of the above analysis, the aim of the current work is to develop and test a new handheld system for analysing the rate of CO_2_ photosynthetic assimilation in plant leaves, based on the accumulative principle of measuring changes in CO_2_.

## 2. Results

### 2.1. General Working Principle of the Developed System

**Figure 1:**
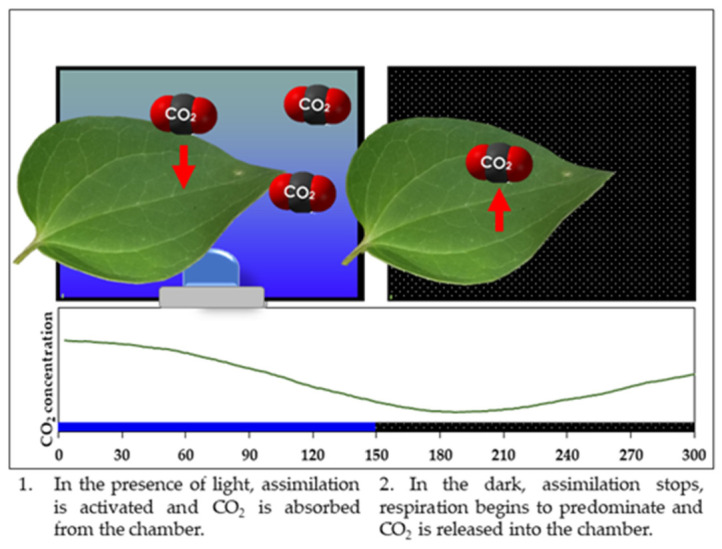
The general working principle of the developed system for measuring photosynthetic CO_2_ assimilation in leaves. The measurement procedure included two main stages: measuring the rate of decline in CO_2_ concentration under light conditions and measuring the rate of increase in CO_2_ concentration in the dark. The rate of photosynthetic CO_2_ assimilation was estimated based on the sum of these values (see Section 2.2 for details).

The system developed used the accumulation principle to measure the rate of photosynthetic CO_2_ assimilation by a plant leaf. In this setup, an object (a plant leaf or a section of it) was sealed inside a closed chamber, one side of which contained a source of blue actinic light; the initial CO_2_ concentration in the chamber corresponded to the CO_2_ content in the ambient atmosphere. The use of blue light, as shown in Supplementary Materials, Figure S1, resulted in sufficient activation of photosynthesis to record the assimilation rate over a short period of time; at the same time, no noticeable activation of transpiration was detected. Based on this, blue light was used to activate photosynthesis in all experiments. When the light was switched on, photosynthesis was activated in the plant leaf and CO_2_ was absorbed, causing the CO_2_ concentration in the measuring chamber to gradually decrease (Figure 1). After a set time interval, which in this study was 150 s (this interval was sufficient for the CO_2_ concentration to change by tens of ppm or more; at the same time, the system is capable of carrying out measurements lasting up to half an hour for each phase), the actinic light was switched off. In the dark, the concentration of CO_2_ began to rise due to the release of CO_2_ by the leaf as a result of respiration; the duration of the dark measurement was also 150 s in the present study. Photosynthetic CO_2_ assimilation was determined as a function of the sum of the rate of CO_2_ uptake in the light and the rate of CO_2_ release in the dark (see Section 2.2 for details). Furthermore, the size of the measurement chamber was sufficiently small to ensure a high leaf area-to-chamber volume ratio (S_ch_/V_ch_); this allowed for an increase in the rate of CO_2_ concentration changes associated with assimilation.

**Figure 2:**
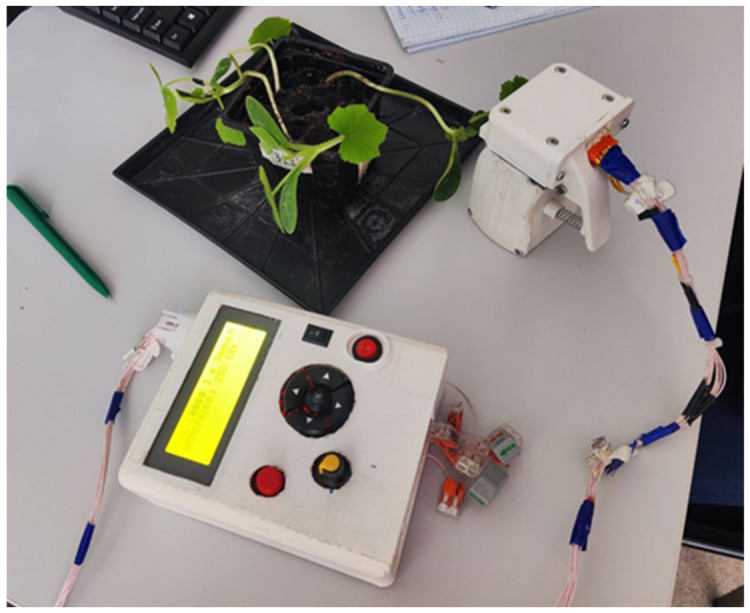
A photograph showing an example of the proposed system’s prototype being used to measure the rate of photosynthetic CO_2_ assimilation in the leaves of 18-day-old pumpkin seedlings.

Figure 2 shows a photograph of the system prototype in use. The developed prototype system consists of a main unit and a measuring unit (see Section 4.1 for more details). Briefly, the main unit includes a board with microcontrollers that control the CO_2_ sensors and collect and record data. The main unit’s housing houses a control panel that allows for parameters (such as measurement time) to be set and the light intensity to be adjusted. The measuring unit is designed like a clamp. The object being studied is clamped in a small chamber on both sides. The outputs of the infrared CO_2_ sensors are located above the object, while a blue actinic light source is located below the object.

### 2.2. Algorithm for Calculating the Rate of Assimilation

**Figure 3:**
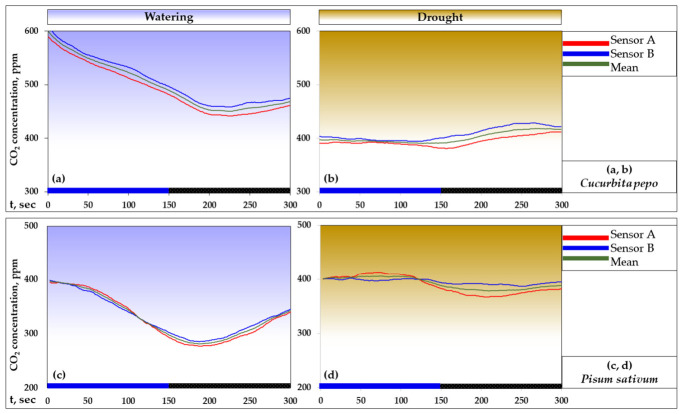
Examples of changes in CO_2_ concentration in measurement chamber measured by the developed prototype under illumination and subsequent darkening. (**a**,**b**) Measurements of the leaves of the pumpkin *Cucurbita pepo* L. under irrigated and drought conditions, respectively; (**c**,**d**) recordings of the leaves of *Pisum sativum* L. under irrigated and drought conditions, respectively. Blue (*x*-axis) indicates illumination with blue actinic light (150 s); black indicates subsequent darkening (150 s). To measure CO_2_ concentration in the prototype, two identical sensors were used simultaneously to record the concentration in the measuring chamber (Sensor A and Sensor B); the mean of the measured values was calculated automatically.

As shown in Figure 3, under conditions of adequate watering, the typical pattern of CO_2_ concentration changes for pumpkin and peas included two phases: a decrease in CO_2_ concentration under light conditions and an increase in this concentration under dark conditions (Figure 3a,c). This curve was fully consistent with theoretical expectations, as under light conditions, CO_2_ uptake during photosynthesis predominates in plants, whilst under dark conditions, CO_2_ release during respiration predominates [39]. With the development of the drought, which was expected to lead to a reduction in photosynthetic assimilation due to stomatal closure [40], the intensity of the decrease in CO_2_ concentration under light and its increase in the dark diminished in the leaves of both plants studied (Figure 3b,d).

**Figure 4:**
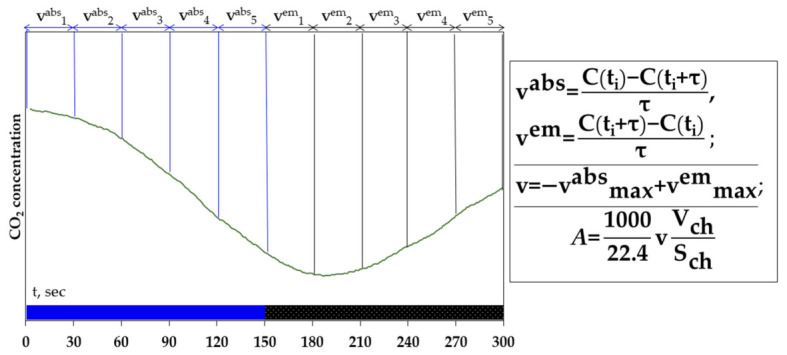
Algorithm for data processing and calculation of the CO_2_ assimilation rate. The green line shows the average CO_2_ concentration changes within the measurement chamber recorded on a pea leaf, calculated between Sensors A and B at each point. The blue line (*x*-axis) shows the duration of the actinic blue light (150 s), and the black line shows the subsequent switching off of the light (150 s). Arrows indicate the start and end of the time intervals (30 s) for which v^abs^_i_ (ppm s^−1^)—the rate of decrease (absorption) in CO_2_ concentration over the time interval from t_i_ to t_i_ + τ—and v^em^_i_ (ppm s^−1^)—the rate of increase (emission) in CO_2_ concentration over the time interval from t_i_ to t_i_ + τ—were calculated; t_i_—beginning of the interval, τ—length of the interval (30 s in this work). C(t_i_) and C(t_i_ + τ)—CO_2_ concentration in the beginning and the end of the interval (ppm), ${v^{abs}}_{max}$—maximal CO_2_ absorption rate under light, ${v^{em}}_{max}$—maximal CO_2_ emission rate in the dark, v—total rate of change in CO_2_ concentration (ppm s^−1^), A—CO_2_ assimilation rate (µmol m^−2^s^−1^), $\frac{V_{ch}}{S_{ch}}$—the ratio of the chamber volume to the area of the leaf in it, $\frac{1000}{22.4}$—the conversion factor from litres to m^3^.

Based on the shape of the obtained curves, it was assumed that the rate of photosynthetic CO_2_ assimilation was equal to the sum of the rate of CO_2_ release under light and the rate of its uptake in the dark, which can be calculated from the derivatives of the CO_2_ concentration change curve. However, determining these velocities was complicated by the presence of lag phases in the CO_2_ concentration curves (after the light was switched on and after it was switched off), as well as by the varying rates of change across different sections of the curves. To account for these features, a special algorithm was developed to calculate the rate of photosynthetic CO_2_ assimilation (Figure 4). In accordance with this, the measured record of CO_2_ concentration changes in the measuring chamber containing a plant leaf was divided into equal time intervals (τ): in this study, these were 30 s. For each of the intervals measured under the light, the rate of CO_2_ concentration decrease (v^abs^) was calculated and the rate for the maximal absolute value (v^abs^_max_) was determined. For each of the intervals measured in the dark, the rates of increase in CO_2_ concentration (v^em^) were calculated and the maximum rate (v^em^_max_) was determined. For each interval, v^abs^ or v^em^ was calculated as a difference in CO_2_ concentrations at the end and the beginning of the interval (C(t + τ) − C(t), where t is the start time of the interval). After that, –v^abs^_max_ and v^em^_max_ were then summed to obtain a value proportional to the total rate of photosynthetic CO_2_ assimilation (v). The total rate of photosynthetic CO_2_ assimilation (*A*) was calculated by multiplying v by the ratio of the volume to the area of the measuring chamber (V_ch_/S_ch_) and by a coefficient ensuring the correct change in units (1000/22.4, where 22.4 is molar volume of gas, l/mol; 1000 is the conversion factor from litres to m^3^).

For automatic calculations, software was developed in C++. The software read the data.txt file, which was generated on an SD card by the developed prototype and contained the dynamics of CO_2_ concentration changes in the measurement chamber (with a time step of 1 s). A filter was applied to the dynamics analysis, replacing a data point in the record with the previous one if the difference between the current point and the previous one exceeded n ppm; such a filter allows the elimination of artefacts arising during the operation of the device (outliers in the record). In the present study, a value of n equal to 40 ppm was used (a change of 40 ppm per second due to physiological processes was impossible, and therefore such changes were an artefact). Furthermore, the programme calculated the rate of photosynthetic CO_2_ assimilation in accordance with the described algorithm. If a single data file contained multiple records separated by column headers, the software generated A values for each record separately. The resulting assimilation values were written to a separate text file, which was then analysed using standard MS Excel tools.

### 2.3. Testing of the Prototype of the Proposed System

**Figure 5:**
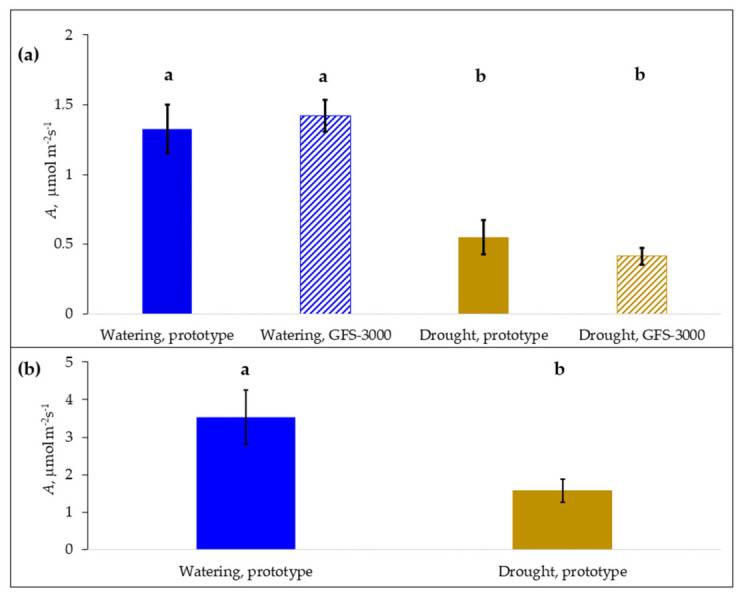
Rates of photosynthetic CO_2_ assimilation (*A*). Different letters indicate statistically significant differences (*p* < 0.05). (**a**) *A*, measured using the developed prototype and a commercial GFS-3000 gas analyser on the leaves of 21-day-old pumpkin plants under conditions of adequate irrigation and a 7-day drought (n = 10); illumination rate: 300 µmol m^−2^s^−1^ 460 nm actinic light. (**b**) *A*, measured using the developed prototype on pea leaves under conditions of adequate irrigation and a 7-day drought (n = 7); illumination rate: 500 µmol m^−2^s^−1^ 460 nm actinic light.

Firstly, to test the prototype of the developed handheld system for measuring photosynthetic CO_2_ assimilation, leaves from 21-day-old pumpkin plants were used. Measurements were carried out on the same leaves using both the prototype and a commercial GFS-3000 gas analyser (Heinz Waltz GmbH, Effeltrich, Germany), which employed a continuous-flow CO_2_ measurement method (Figure 5a).

**Figure 6:**
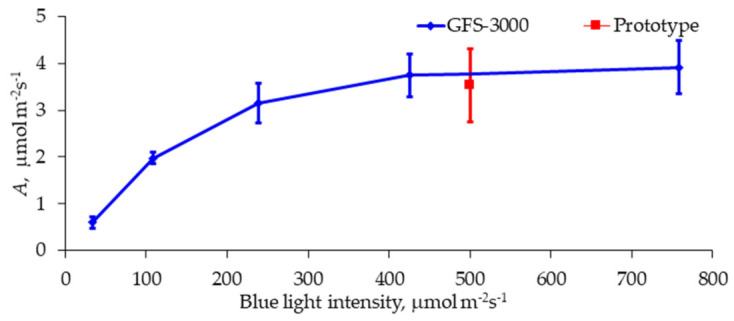
Comparison of photosynthetic assimilation rates obtained using the commercial GFS-3000 system and a prototype of the proposed system. The blue line shows the curve of the dependence of photosynthetic assimilation rate in pea leaves at different intensities of blue actinic light, obtained using a combination of the GFS-3000 gas analyser with the Gas exchange cuvette 3010-Dual measuring chamber and the Dual-PAM-100 fluorometer with a standard light source. The red point shows the assimilation rate in pea leaves, obtained using the prototype of the proposed system at a light intensity of 500 µmol m^−2^s^−1^.

Secondly, to test the device’s applicability to other plant species, the effect of drought on photosynthetic CO_2_ assimilation in 14-day-old pea leaves was investigated using the developed prototype (Figure 5b). A comparison of the assimilation rate obtained on the prototype and commercial system (GFS-3000) on peas was also carried out. To compare the results of these measurements with CO_2_ assimilation measurements using the developed prototype, light-dependent relationships between the CO_2_ assimilation rate and light intensity were obtained during the study (Figure 6).

## 3. Discussion

The development and improvement of systems for measuring photosynthesis parameters is an important area of applied research; in particular, instruments for measuring the rate of photosynthetic CO_2_ assimilation are being actively developed [28,29]. Modern high-cost systems for measuring *A* (e.g., Li-6800, GFS-3000, CIRAS-4, LCA4, etc.) provide a continuous air flow around the object under study (e.g., a plant leaf) and measure CO_2_ changes in such a flow, which requires the use of high-precision infrared CO_2_ sensors, air pumps, air flow meters, and other units [28,29]. The development of simpler and more accessible low-cost systems for measuring photosynthetic assimilation is an important challenge for modern instrument engineering. The current work is devoted to its solution.

The system proposed in this work employs an accumulative measurement principle to assess changes in CO_2_ concentration for the purpose of evaluating photosynthetic CO_2_ assimilation [32,35,36,37,38]. The transition from continuous recording of CO_2_ concentration in the air flow passing around the leaf (modern commercial systems) to long-term (~several minutes) measurements of changes in CO_2_ concentration in a measurement chamber containing the leaf makes it possible to significantly increase the amplitude of such changes. This allows the use of simple infrared sensors for measuring CO_2_ concentration, which have a sufficiently high error rate, in the developed handheld system. The accumulative operating principle also makes it possible to reject the moving parts of the gas analyser (primarily the pump maintaining the air flow and the units associated with measuring such a flow), which simplify the system’s design and reduce its mass. Finally, the large amplitude of CO_2_ concentration changes when implementing the accumulative approach, combined with the use of blue actinic light—which is effective in stimulating stomatal opening [41] and, as shown in this work, is capable of photosynthesis activation (Supplementary Materials, Figure S1)—reduces the influence of ambient CO_2_ concentration on the measurements (this effect is particularly pronounced during measurements in enclosed spaces). This makes it possible to eliminate the use of consumable reagents (air dryers and humidifiers, CO_2_ absorbers and sources) during measurements, despite the fact that such reagents are widely used for measuring plant gas exchange using commercial systems.

The noted features significantly simplify the system, reduce its weight (<1 kg for the prototype), and lower its manufacturing cost.

Testing of the developed system’s prototype shows that the values of photosynthetic CO_2_ assimilation measured in pumpkin and pea using this system are similar to the assimilation values measured under comparable light intensities using a commercial GFS-3000 gas analyser, which is a precise instrument for studying plant assimilation and transpiration [40]. The deviations between the mean photosynthetic CO_2_ assimilation values obtained with the developed prototype and the commercial gas analyser did not exceed 0.15 µmol m^−2^ s^−1^ for pumpkin and 0.25 µmol m^−2^ s^−1^ for pea. Under sufficient watering conditions, the relative difference between these measurements was no more than 6–7%.

Additionally, for both peas and pumpkin, drought statistically significantly reduces the rate of photosynthetic CO_2_ assimilation (by approximately 2.5 times), which is fully consistent with the literature data on the suppression of photosynthetic processes under water-deficient conditions, associated, in particular, with stomatal closure and a reduction in the CO_2_ flux into the leaf and, further, into the chloroplast stroma [42].

Thus, our results show that the developed prototype of a handheld system for measuring photosynthetic CO_2_ assimilation has sufficient measurement accuracy to detect the assimilation rate under well-watered conditions and a reduction in this rate under the influence of stressors (at least drought) in various plants (pumpkins, peas). It should be noted that such a comparison of two systems does not accurately show the measurement error of the proposed system because commercial flow-through systems are known to exhibit significant differences in measurement results [43,44]. Therefore, our experimental results confirm the applicability of the developed system for measuring rates of photosynthetic CO_2_ assimilation, because the measured photosynthetic CO_2_ assimilation rates are comparable to those obtained with a widely used commercial system (GFS-3000).

Overall, the developed handheld system for measuring photosynthetic CO_2_ assimilation in plant leaves (prototype) measures these assimilation rates under favourable and stressful (drought) conditions. The system is compact and lightweight, does not require a constant air flow (which reduces the likelihood of breakdown due to the absence of moving parts), does not require the use of consumables (air dryers and humidifiers, CO_2_ absorbers and sources) and has low-cost components. It can be expected that, due to these characteristics, the simple system developed may become a significantly more accessible tool compared to standard commercial systems for measuring CO_2_ assimilation in plants, which are currently widely used for laboratory and field studies [28,29].

However, it is important to note that determining photosynthetic CO_2_ assimilation using the developed system requires a relatively long-time interval (300 s in the current study), i.e., such a system is unlikely to be an effective tool for investigating the dynamics of rapid responses in the photosynthetic apparatus (e.g., responses to the propagation of electrical signals, which unfold over tens of seconds and minutes [45,46,47]). It is also likely that the developed system will be of limited use for long-term and continuous measurement of photosynthetic processes (assimilation) on a single leaf, which may be required in certain research tasks [48,49,50]. Furthermore, as a result of the deliberate maximal simplification of the system, its capabilities are exclusively limited to measuring photosynthetic assimilation, while there is no possibility of determining stomatal conductance or H_2_O transpiration. It can, however, be expected that in the case of large-scale screening studies requiring the measurement of the assimilation in a significant number of different leaves, the temporal efficiency of using the developed system and commercial gas analysers will be comparable, as the use of the latter requires at least 5–10 min to perform a new measurement (for example, each new measurement using the GFS-3000 requires the determination of a zero point, reaching target CO_2_ and H_2_O concentrations, and stabilisation of the measured parameters) [51].

Apart from that, it should be noted that the developed system for measuring CO_2_ photosynthetic assimilation in plant leaves could serve as a tool for the rapid detection of stress-induced changes, as photosynthesis is one of the key targets of stressors [17,18]. Moreover, given the simplicity of the measurements and the high mobility of the developed device, not only can the absolute values of photosynthetic CO_2_ assimilation be used to identify stressors, but also the differences (or dispersion) in such assimilation across different parts of the plant, as it is known that the effect of stressors on a plant organism typically develops unevenly across space, leading to heterogeneous changes in photosynthetic activity [52,53].

## 4. Materials and Methods

### 4.1. Scheme of the Developed System

**Figure 7:**
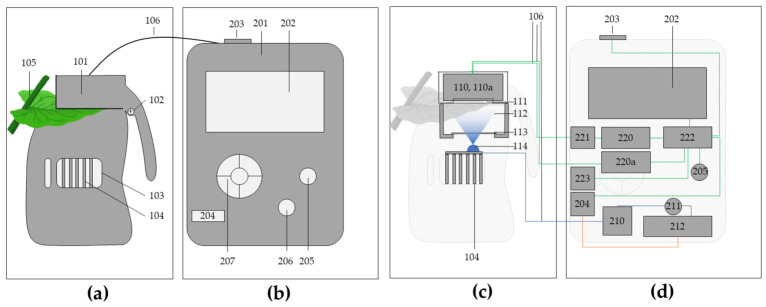
Schematic diagram showing the external appearance of the measurement (**a**) and main (**b**) modules of the developed prototype of a handheld analyser for measuring CO_2_ photosynthetic assimilation in plant leaves. (**c**,**d**) show the block diagram of the measuring (**c**) and main (**d**) modules of the developed prototype of the photosynthetic CO_2_ assimilation analyser for plant leaves, including its main components. The system for measuring photosynthetic CO_2_ assimilation in plant leaves shown in (**a**–**d**) consists of: (**a**,**c**), the system’s measurement module; 101, the housing of the non-flow chamber with a specimen clamp, made of ABS plastic; 102, a spring-loaded clip-type fastener for the upper part of the chamber; 103, the window of the illuminator radiator; 104, the illuminator radiator; 105, the intended position of the test specimen; 106, the cable connecting modules 1 and 2; 110, 110a, CO_2_ infrared sensors—two MH-Z1311A sensors (Zhengzhou Winsen Electronics Technology Co., Ltd., Zhengzhou, China); 111, upper half of the non-flow-through chamber with sensor measurement port outlets, volume 691.75 mm^3^; 112, lower half of the non-flow-through chamber with a volume of 1470.875 mm^3^; 113, cover glass for the microscope, 18 × 18 mm, 0.17 mm thick; 114, TDS-P003L4C04 LED (blue LED, 140°, 460 nm, 40 lm, 3 W (TDS Lightning Co., Ltd., Wuxi, China); (**b**,**d**), main system module; 201, main module housing, made of PLA plastic; 202, LCD2004 liquid crystal display (Shenzhen Chengsuchuang Technology Co., Ltd., Shenzhen, China); 203, module for reading and saving data, reading/writing to SPI microSD memory cards; 204, on/off button; 205, start and stop recording button; 206, potentiometer control; 207, navigation buttons for device configuration with a central ‘OK’ button; 210, DC/DC converter; 211, the potentiometer itself; 212, battery, LiPo 3S 11.1V, 3300 mAh, 60C, T-Plug (Teranty, Guangzhou, China); 220, 220a, microcontrollers to ensure sensor functionality, 2 ATmega328 microcontrollers with CH340 USB driver chips (Shenzhen Chengsuchuang Technology Co., Ltd., Shenzhen, China); 221, board with control relays; 222, microcontroller for managing data storage and recording, and system operating modes: ESP-32-WROOM-32 with Bluetooth and Wi-Fi (Espressif, Shanghai, China); 223, control button board.

Figure 7 shows a schematic representation of the external appearance of the modules of the developed prototype of a handheld analyser for measuring CO_2_ photosynthetic assimilation in plant leaves, which operates on the principle of accumulative measurement, as well as a block diagram of the prototype, including a description of its components and their specific brands.

The main module case was created using 3D printing technology from ABS plastic, incorporating ventilation openings for the heat sink no larger than 20 × 20 mm. The case with a clamp for the test object, measuring 100 mm × 64 mm × 80 mm, formed a chamber consisting of two parts: on one side were two MH-Z1311A CO_2_ infrared sensors (Zhengzhou Winsen Electronics Technology Co., Ltd., Zhengzhou, China), whose inlets were oriented towards the test object and, during measurement, abutted it on one side, forming the upper half of a chamber with a volume of 691.75 mm^3^, isolated from the surrounding environment. On the other side, an illuminator positioned beneath a 1470.875 mm^3^ cavity—enclosed by an 18 × 18 mm microscope cover glass, 0.17 mm thick—provided adjustable actinic illumination in the blue spectrum. A TDS-P003L4C04 LED (140° blue LED, 460 nm, 40 lm, 3W (TDS Lightning Co., Ltd., Wuxi, China)) achieved an actinic illumination range of 0–750 μmol m^−2^s^−1^ at a distance of 2 cm between the illuminator and the object under study; an aluminium heat sink is also located beneath the LED in the system to ensure the illuminator is cooled.

The main control and data processing module comprised a casing made of polymer and other materials, which was created using 3D printing technology with PLA plastic. The dimensions of the main module are 60 mm × 145 mm × 185 mm. The module contains the main control elements of the system, connected to the measuring module via wires. The main module contains two Atmega328 CH340 microcontrollers (Shenzhen Chengsuchuang Technology Co., Ltd., Shenzhen, China), which control each of the CO_2_ sensors; as well as an ESP-32-WROOM-32 microcontroller (Espressif, Shanghai, China), connected to the Atmega328 CH340, which acts as a central data exchange point for the sensors and also stores the device settings; other electronic components, including a relay unit to monitor sensor operation, as well as a character display on an LCD2004 liquid crystal matrix (Shenzhen Chengsuchuang Technology Co., Ltd., Shenzhen, China); buttons for controlling settings, including on/off and record start buttons; a module for reading and saving data, for example, an SPI microSD card read/write module; as well as a rheostat required to regulate the brightness of the LED in the measurement module, connected in series between the DC/DC converter for converting the 12 V input power to 5 V and the positive 12 V power input. The system’s autonomous operation is achieved through the use of a removable and rechargeable battery (LiPo 3S 11.1V, 3300 mAh, 60C, T-Plug (Teranty, Guangzhou, China)).

### 4.2. Growing Plant Material

To carry out the study, 21-day-old pumpkin plants (*Cucurbita pepo* L., ‘Tsukesha’ variety) and 14-day-old garden pea plants (*Pisum sativum* L., ‘Falensky Yubileiny’ variety) were used. The seeds were germinated in containers with water and filter paper, after which the seedlings were transplanted into pots containing soil. All plants were grown in a growing room with stable conditions (+23 °C, 16 h full-spectrum lighting, PPFD ~50 µmol m^−2^s^−1^) in ‘Peter peat’ universal peat-based potting mix (Peter peat LLC., Dzerzhinsky, Russia), with four plants per pot. Plants in the control group were watered regularly, three times a week. Plants from the experimental group were exposed to artificial drought conditions by cutting off watering 7 days before the start of measurements. The sample size was as follows: 10 watered pumpkin plants and 10 drought-stressed pumpkin plants; 7 watered pea plants and 7 drought-stressed pea plants.

### 4.3. Recording the Rate of Photosynthetic Assimilation Using a Prototype of the Proposed System

Before the experiment, the plants were kept under background laboratory lighting conditions identical to the lighting during cultivation and did not undergo dark adaptation.

Operation with the prototype of the proposed system was carried out in the following order: a leaf of the plant under study was clamped in the measuring chamber (see Figure 2) without detaching it from the plant. As soon as the sample was placed in the chamber, recording of CO_2_ concentration dynamics began, with the blue actinic light being switched on and off in sequence; each measurement phase lasted 150 s.

In the case of measurements on pumpkin plants, the examined leaf occupied the entire chamber, so the known chamber area was used to calculate the assimilation rate. For the measurements on pea leaves, whichever was smaller—the area of the measuring chamber or the area of the leaf being measured, estimated using the ‘ImageJ 1.53k’ software (National Institutes of Health, Bethesda, MD, USA) [54]—was used to calculate the assimilation rate.

The intensity of the actinic blue light used in the measurements was 300 µmol m^−2^s^−1^ for pumpkin and 500 µmol m^−2^s^−1^ for peas [55,56]. Calibration of light intensity at a wavelength of 460 nm was carried out using the PM100D light flux meter with S120C sensor (Thorlabs Ultrafast Optoelectronics, Ann Arbor, MI, USA). All measurements were carried out under laboratory conditions at a temperature of +23 °C.

### 4.4. Measurement of Photosynthetic Assimilation and Transpiration Rate Using a Commercial Equivalent (GFS-3000)

On pumpkin plants, measurements of the photosynthetic assimilation and transpiration rate were carried out using a GFS-3000 gas analyser, equipped with a standard 3010-S measuring chamber suitable for large leaves (Heinz Waltz GmbH, Effeltrich, Germany). All measurements were carried out under laboratory conditions at an air temperature of +23 °C and a cuvette temperature of +23 °C. When comparing the GFS-3000 and the prototype of the proposed system on pumpkin leaves, the same leaves were used with an interval of 30 min between measurements.

A custom-made illuminator was used to illuminate the leaf and activate photosynthesis, comprising a TDS-P003L4C04 LED (blue LED 140°, 460 nm, 40 lm, 3 W (TDS Lightning Co., Ltd., Wuxi, China)) and an LF-AAD030-0750-42 LED driver (Lifud Technology Co., Ltd., Shenzhen, China). The standard light source for the 3010-S measuring head was not used, since it has spectral characteristics that do not correspond to those of the illuminator built into the prototype of the proposed system, while the purpose of the experiment was to compare measurements on the prototype and the GFS-3000, carried out under equal conditions. Calibration of light intensity at a wavelength of 460 nm was carried out using the PM100D light flux meter with S120C sensor (Thorlabs Ultrafast Optoelectronics, Ann Arbor, MI, USA). As in the proposed system, the LED was positioned below the measurement chamber and illuminated the abaxial side of the leaf being measured. The light intensity during measurement with the GFS-3000 gas analyser was equal to the light intensity used in measurements with the developed prototype system for measuring CO_2_ photosynthetic assimilation (300 µmol m^−2^s^−1^).

To simulate the recording protocol used in the proposed system when measuring the assimilation rate in pumpkin plants using the GFS-3000, readings were first taken from the leaf placed in the chamber in the light for 150 s, and then for 150 s in the dark. The custom-made illuminator was used, and leaf illumination was provided through a standard window in the measuring chamber. Points with the maximum recorded assimilation rate were selected for analysis. When comparing the assimilation rates recorded using the prototype of the proposed system and the GFS-3000, plants were selected at random for sequential measurement on one of the systems, after which the CO_2_ assimilation rate was measured on the other system following a 30 min adaptation interval.

For pea plants, the assimilation rate was recorded using the GFS-3000 gas analyser. As pea leaves have a relatively small surface area, studying them in the standard 3010-S measurement chamber for the GFS-3000 using the prototype’s light source was not optimal: the area of the clamped region in the chamber (8 cm^2^) was significantly larger than the average area of a pea leaf in our experiments (~2.65 cm^2^). To overcome this limitation, the standard combination of the GFS-3000 gas analyser, the Dual-PAM-100 PAM fluorometer and the 3010-Dual gas exchange cuvette was used to determine the light dependence of CO_2_ photosynthetic assimilation on the intensity of blue actinic light. In this case, the standard Dual-PAM-100 light source was used as the illumination source. In a comparison of the GFS-3000 and the prototype of the proposed system on pea leaves, leaves of different plants were used; however, the leaf numbers and age of the plants were identical.

### 4.5. Statistical Analysis

The study was conducted on individual plants; the number of independent replicates is shown in the figures. Means, standard deviations of the mean and typical examples are shown in the figures. To assess the statistical significance of differences, Student’s *t*-test for paired samples was used. Calculations were performed with MS Excel 2021 (Microsoft Inc., Redmond, WA, USA).

## 5. Conclusions

The alternative approach proposed in this work, based on analysing accumulative changes in CO_2_ concentrations within a compact measuring chamber that isolates a section of the leaf (or a small leaf), demonstrated its effectiveness. The results of comparison of the commercial analogue and developed prototype showed that the accumulative method may be used as a base for future device development, since, firstly, the measured values of the assimilation rates corresponded to each other with little deviation, and secondly, the experiments demonstrated the ability of the prototype to sense stress changes in photosynthetic processes in the plant.

It should be emphasised that the developed analyser of photosynthetic CO_2_ assimilation in plant leaves has the potential to become a promising tool both for addressing applied challenges in agriculture and environmental monitoring (rapid assessment of biological productivity based on assimilation, early diagnosis of stress-induced changes), as well as for scientific research in the fields of plant physiology and biophysics. The compactness, simplicity and low cost of the developed system opens up prospects for its implementation in laboratory and field practice, including the educational process and precision farming monitoring systems.

## 6. Patents

Following the completion of the work, an application was filed for the registration of invention (date of application acceptance 26.09.2025, application No. 2025127770) with the Federal Service for Intellectual Property, Russian Federation.

## Data Availability

The original contributions presented in this study are included in the article/Supplementary Materials. Further inquiries can be directed to the corresponding author.

## Supplementary Materials

The following supporting information can be downloaded at: https://www.mdpi.com/article/10.3390/plants15121888/s1, Figure S1. An example of a recording of the assimilation rate dynamics obtained on a pumpkin leaf (normal watering) using the GFS-3000 (Heinz Waltz GmbH, Effeltrich, Germany).

## Abbreviations

The following abbreviations are used in this manuscript:

PAM	Pulse Amplitude Modulation
PPFD	Photosynthetically Active Photon Flux Density
LED	Light-emitting diode
