# Supplementary material for: Development of a New Handheld Device for Measuring Photosynthetic Carbon Dioxide Assimilation in Plant Leaves
Source: Plants (Basel). 2026 Jun 18;15(12):1888. doi: 10.3390/plants15121888 (PMC13307541; doi:10.3390/plants15121888)
Supplement: Supplementary file 1 [file plants-15-01888-s001.zip › plants-4346297-supplementary.pdf]

Supplementary materials

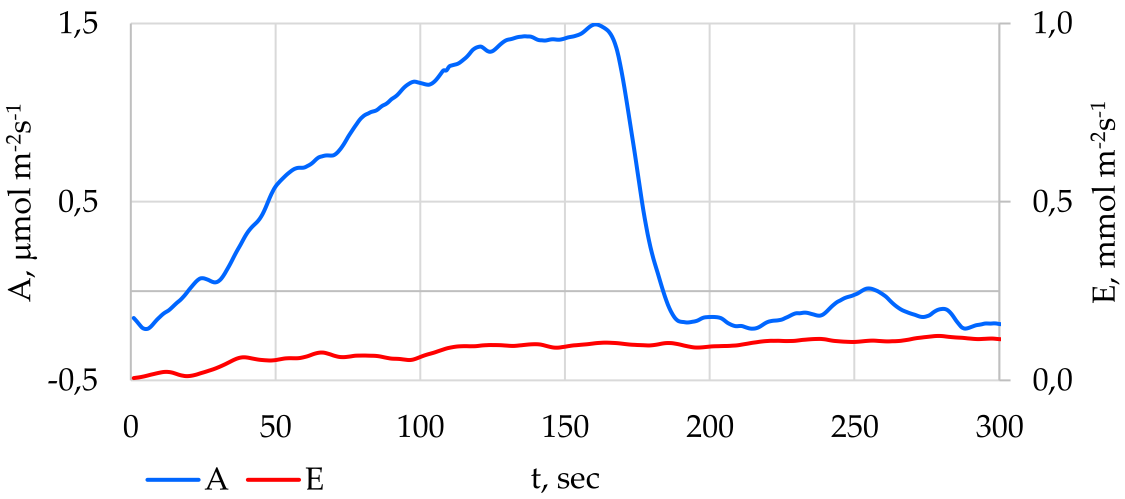

Figure S1. An example of a recording of the assimilation rate dynamics obtained on a pumpkin leaf (normal watering) using the GFS-3000 (Heinz Waltz GmbH, Effeltrich, Germany). The blue curve reflects changes in the leaf's assimilation rate (*A)* during sequential switching on (blue timeline) and switching off (black timeline) of blue actinic light. The red line reflects dynamics of transpiration rate (*E*); discrepancy in the forms of the records, and in general small changes in transpiration rate *E*, reflecting stomatal conductance, indicate that when using blue actinic light, it is the activation of photosynthesis that occurs, but not a change in stomatal activity.
